# Synthesis of Cu–Mo/TiO_2_ and Co–Mo/TiO_2_ photocatalysts for the efficient degradation of organic pollutants in water

**DOI:** 10.3762/bjnano.17.37

**Published:** 2026-04-27

**Authors:** Ilse Acosta, Brenda Zermeño, Edgar Moctezuma, Luis F Garay-Rodríguez, Isaías Juárez-Ramírez

**Affiliations:** 1 Facultad de Ciencias Químicas, Universidad Autónoma de San Luis Potosí, Av. Manuel Nava # 6, San Luis Potosí, S.L.P., 78290, México; 2 Departamento de Ecomateriales y Energía, Facultad de Ingeniería Civil, Universidad Autónoma de Nuevo León, Ciudad Universitaria, San Nicolás de los Garza, N.L., 66455, Mexico

**Keywords:** co-doping, photocatalysis, titanium dioxide, water remediation

## Abstract

Co-doped titanium dioxide materials were successfully synthesized by the sol–gel method. Molybdenum was incorporated into all materials at 0.5 wt %, while the co-dopants, copper and cobalt, were added at 0.2–0.5 wt %. The co-doped TiO_2_ photocatalysts were characterized by XRD, SEM, N_2_ physisorption, UV–vis diffuse reflectance spectroscopy, and photoluminescence spectroscopy. The structural characterization showed stabilization of the anatase phase, and lattice distortion was evidenced after dopant incorporation into the TiO_2_ structure. Morphological characterization showed poorly defined spherical particles that decreased in size with increasing Cu and Co concentrations. PL spectra showed an additional signal attributed to the ability of the metal dopants to capture electrons. The point of zero charge of the photocatalytic systems TiO_2_, Cu–Mo/TiO_2_, and Co–Mo/TiO_2_ was evaluated and reported. Materials with lower concentrations of the co-dopants Cu and Co were more efficient at degrading ketoprofen. The most efficient photocatalyst was 0.2 Cu–0.5 Mo/TiO_2_, which achieved the complete degradation of ketoprofen and 90% of mineralization. It was determined that HO^·^ radicals play an important role in the oxidation reactions.

## Introduction

Water is an essential part of every living entity. Unfortunately, water quality is negatively impacted by the increasing population, industrial operations, and agricultural activities. Therefore, it is essential to develop technologies to conserve and remediate contaminated water. A significant environmental challenge in water remediation is the presence of organic contaminants. These products are frequently detected in aquatic bodies due to human use and include pharmaceuticals, pesticides, and industrial chemicals, which significantly degrade drinking water quality [1]. To achieve the efficient removal of emerging pollutants, advanced oxidation processes (AOPs) have been considered an alternative to conventional water treatment technologies [2]. They are initiated through the formation of reactive and short-lived radicals (e.g., ^·^OH, HOO^·^, ^·^O_2_^−^), where the hydroxyl radical (HO^·^) is the most reactive and powerful oxidant (*E*^0^ = 2.7 V), which reacts with most organic compounds [2]. Among AOPs, TiO_2_ photocatalysis is one of the most viable environmental technologies due to its low cost and the stability of TiO_2_. Limitations of TiO_2_ in photocatalysis applications come from rapid charge recombination and the wide bandgap [3]. Various strategies have been developed to overcome these limitations, including doping, noble-metal deposition, heterogeneous structures, and surface sensitization. Doping is a widely employed method to generate impurity states in the forbidden region or to reduce the effective bandgap. Doping TiO_2_ with transition metal ions can adjust the optical bandgap, broaden the light absorption range, and enhance the quantum efficiency [4]. However, the partially occupied impurity states generated can act as recombination centers for photoexcited carriers, leading to band-to-band recombination [3]. To prevent charge recombination effects, the new bands generated by doping can be passivated, and they will not act as charge recombination centers if the semiconductor oxide is co-doped with two different elements [5]. TiO_2_ co-doping can be achieved by incorporating combinations of metal/metal, non-metal/metal, and non-metal/non-metal dopants into the semiconductor matrix. The incorporation of co-dopants results in the formation of heterostructures with different electronic structures compared to the TiO_2_ structure, which promotes charge separation and visible light absorption [6]. The incorporation of two types of cations into the TiO_2_ lattice and the effects on photocatalytic performance have been reported in several studies [7–10]. Crucial factors for successfully co-doping a material are the selection of compatible co-dopants and the synthesis method to introduce the dopants [11]. The main objective of working with metal/metal co-doped TiO_2_ is to use transition metals, which are abundant and relatively cheap. A well-reported strategy is the charge compensation in metal/metal co-doped TiO_2_, which is achieved through the combination of ions with low and with high valences; the substitution by doping ions on the Ti^4+^ sites could be balanced by the doping levels in TiO_6_ octahedra [3]. Mo^6+^ is a transition metal ion with high valence that exhibits exceptional optical, electronic, and catalytic characteristics [12]. There are a few reports about the doping of TiO_2_ with molybdenum [13–16]; it has been shown that Mo can improve light absorption and photocatalytic activity of the material through the generation of oxygen vacancies, which act as electron traps [12]. The Mo^6+^ ion has a radius similar to that of Ti^4+^; thus, it is ideal for introduction into the TiO_2_ lattice without causing significant disturbances. Mo doping introduces a donor level below the conduction band, thereby reducing the semiconductor bandgap. As a disadvantage, the addition of Mo can result in the formation of large crystals, affecting the surface area and the ability to adsorb pollutants. In contrast, Cu^2+^ and Co^2+^ are transition metal ions with low valence that have shown improvements when used as dopants in photocatalytic processes [17–20]. Cu^2+^ introduces shallow trapping sites that prolong charge-carrier lifetime [21]; it also creates defects in the TiO_2_ lattice, which increase the optical absorbance [22]. Cobalt-doped TiO_2_ can promote light absorption and induce lattice distortion and defects [19]. However, there are some challenges that need to be addressed to fully optimize Cu–TiO_2_ and Co–TiO_2_ systems. Although there are reports related to M-doped TiO_2_ (M = Cu, Co, or Mo), and different co-doped TiO_2_ systems, there is no report in the literature about the specific photocatalysts Cu–Mo/TiO_2_ and Co–Mo/TiO_2_. However, due to the promising effects reported and previously described, the use of the metals Cu, Co, and Mo was considered for the synthesis of two photocatalytic systems. The synergetic strategy of co-doping TiO_2_ with the high-valence Mo ions and the Cu or Co ions with low valence can be used to address the shortcomings of individual doping systems to optimize charge transfer and reduce recombination. Metal/metal co-doping has not received much attention; however, it is a promising alternative since the doping of TiO_2_ can be done during its synthesis or through an impregnation technique, which is a very simple method that does not require high energy consumption. In addition, the transition metal precursors are relatively inexpensive, which makes these materials more attractive to be used in environmental remediation processes. This research project reports the synthesis and characterization of two photocatalyst systems not reported before for water remediation, namely, co-doped photocatalysts Cu–Mo/TiO_2_ and Co–Mo/TiO_2_ synthesized by the sol–gel method. The structural, optical, and morphological properties were determined. Finally, the photocatalytic behavior of the materials was studied in the photocatalytic oxidation of ketoprofen (KTP) under UV irradiation, with the aim of studying the charge-transfer improvement. Ketoprofen degradation, adsorption, kinetics, and reaction pathways have been previously studied and reported [23–24]. We chose this medicine as a model molecule since this non-steroidal anti-inflammatory drug is of great environmental relevance as it has been detected in several aquatic bodies.

## Results and Discussion

### Structural characterization

**Figure 1:**
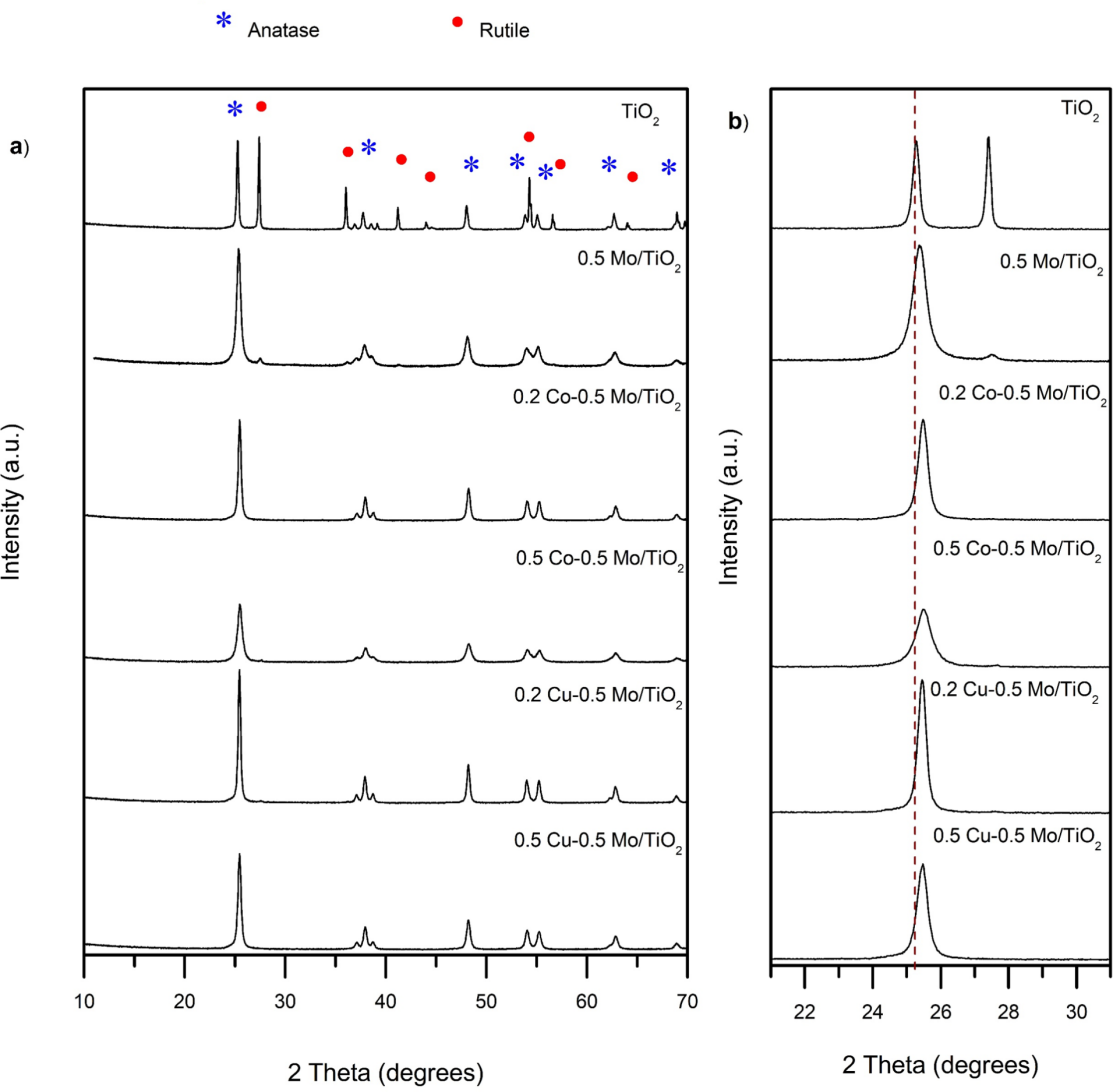
(a) XRD patterns of TiO_2_ and co-doped TiO_2_ materials and (b) diffraction peak shift in TiO_2_ materials.

Figure 1a shows the diffraction patterns of TiO_2_ and the co-doped TiO_2_ materials prepared by the sol–gel method. The sample TiO_2_ shows the crystalline structures anatase and rutile according to the crystallographic cards CPDS 121 and 4031 [25–26], respectively. The composition of each phase was determined using the MAUD software, resulting in 53% and 47% for the anatase and rutile phases, respectively. Figure 1a also shows the diffraction pattern of the 0.5 Mo/TiO_2_ material, which revealed that the Mo in-situ incorporation into the TiO_2_ stabilizes the anatase phase. All co-doped TiO_2_ materials exhibit diffraction peaks corresponding only to the anatase phase, with a preferential orientation along the (101) plane. The photocatalytic activity of both anatase and rutile crystalline phases has been widely discussed in the literature, and it is generally accepted that anatase exhibits higher photocatalytic activity due to its higher oxidation potential and better surface properties [27]. It is expected that the structural properties of the co-doped materials will promote the photocatalytic oxidation reaction.

A decrease in the intensity of the diffraction peaks and the appearance of wider peaks were observed with increasing Cu and Co content. No evidence of diffraction peaks attributable to molybdenum, copper, or cobalt oxides was observed. If metal oxides are present in the material, they are small, highly dispersed crystals that the technique cannot detect. Figure 1b shows the materials’ diffractogram in the range of 2θ = 21–31°. It can be observed that, upon incorporation of metallic ions, the signal at 25.2° shows a slight shift towards higher 2θ values in all the co-doped materials. This indicates the distortion of the anatase crystalline network due to the addition of dopants.

Table 1 reports the cell parameters and cell volumes determined from the anatase diffraction peaks using the MAUD software. A slight increase in the cell volume is observed for the modified titanium oxides. Since molybdenum can be present in the oxidation state Mo^6+^, presenting an ionic radius of 0.62 Å, whereas, Ti^4+^ presents an ionic radius of 0.74 Å [5], the distortion of the crystal lattice may be a consequence of the insertion of the Mo with a smaller ionic radius. The increase of the unit cell volume could be attributed to the electronegativity differences between Mo and Ti, reflected in the larger Ti–Mo distance, compared to the Ti–Ti distance of undoped TiO_2_ [28]. Mo^6+^ can substitute the Ti^4+^ atom into the crystalline network. Another possibility is the Mo^4+^ or Mo^6+^ interstitial doping. However, due to energy issues, substitution doping is the most common and likely [29]. Further, the presence of Mo as a dopant is evident since it causes the inhibition of crystalline phase transformation from anatase to rutile, which is an expected behavior for cationic dopants of valence higher than 4. The change in lattice parameters may also indicate the presence of oxygen vacancies created by the incorporation of impurities as Mo. The charge compensation could be mainly achieved by the ionized vacancies, especially by doubly ionized oxygen vacancies [29]. In addition, the volume cell increase was observed not only in the material Mo/TiO_2_, but also in the materials with the content of the two metal ions, which may indicate a slight contribution from these to the lattice distortion. Additionally, the crystallite size of the materials was determined using the Debye–Scherrer equation and the results are reported in Table 1. The presence of Mo, Cu, and Co metal ions decreases the crystallite size. The inhibition of grain growth in the crystal lattices of the materials may be due to the decrease in the number of intergranular contacts between neighboring grains of titania as the amount of dopant in TiO_2_ increases [5].

**Table 1:** Cell parameters and crystallite sizes calculated for the co-doped TiO_2_ materials using the TiO_2_ anatase phase.

Material	Cell parameters					Crystallite size (nm)
	*a* (Å)	*b* (Å)	*c* (Å)	*V* (Å^3^)	*R*_wp_/*R*_exp_	

TiO_2_	3.784	3.784	9.521	136.332	1.67	32.965
0.5 Cu/TiO_2_	3.789	3.789	9.512	136.559	1.88	26.972
0.2 Cu–0.5 Mo/TiO_2_	3.786	3.786	9.524	136.570	1.55	25.939
0.5 Cu–0.5 Mo/TiO_2_	3.787	3.787	9.521	136.566	1.40	19.974
0.2 Co–0.5 Mo/TiO_2_	3.787	3.787	9.523	136.591	1.52	22.254
0.5 Co–0.5 Mo/TiO_2_	3.788	3.788	9.520	136.680	1.39	13.405

To confirm the presence of copper, cobalt, and molybdenum in the TiO_2_ materials, XPS analysis was performed. For the 0.5 Cu–0.5 Mo/TiO_2_ and 0.5 Co–0.5 Mo/TiO_2_ samples, Ti 2p_3/2_ and Ti 2p_1/2_ peaks are observed at 459.1 and 464.9 eV, respectively (Supporting Information File 1), indicating that Ti exists predominantly in the Ti^4+^ oxidation state [30]. The samples also show the Mo 2d_5/2_ and 2d_3/2_ peaks at 232.6 and 235.9 eV, respectively, indicating the Mo^6+^ state [14]. In addition, the sample 0.5 Cu–0.5 Mo/TiO_2_ presents the Cu 2p peaks at 932.5 and 952.4 eV, which indicate the presence of reduced copper species that can be attributed to either Cu–O–Ti bonds, or the presence of Cu^1+^ species [18]. Finally, the sample 0.5 Co–0.5 Mo/TiO_2_ shows the Co 2p_3/2_ and Co 2p_1/2_ peaks at 781.4 and 797.2 eV, respectively [31]. The in situ incorporation of molybdenum during sol–gel synthesis results in the stabilization of the anatase crystalline phase and network distortion, indicating that Mo acts as a lattice dopant. In contrast, the addition of Cu and Co to the TiO_2_ material by the impregnation technique, as well as the identification of the metallic species by the surface analytical technique, XPS, suggests that the TiO_2_ surface is modified by these species.

### Morphological and textural characterization

**Figure 2:**
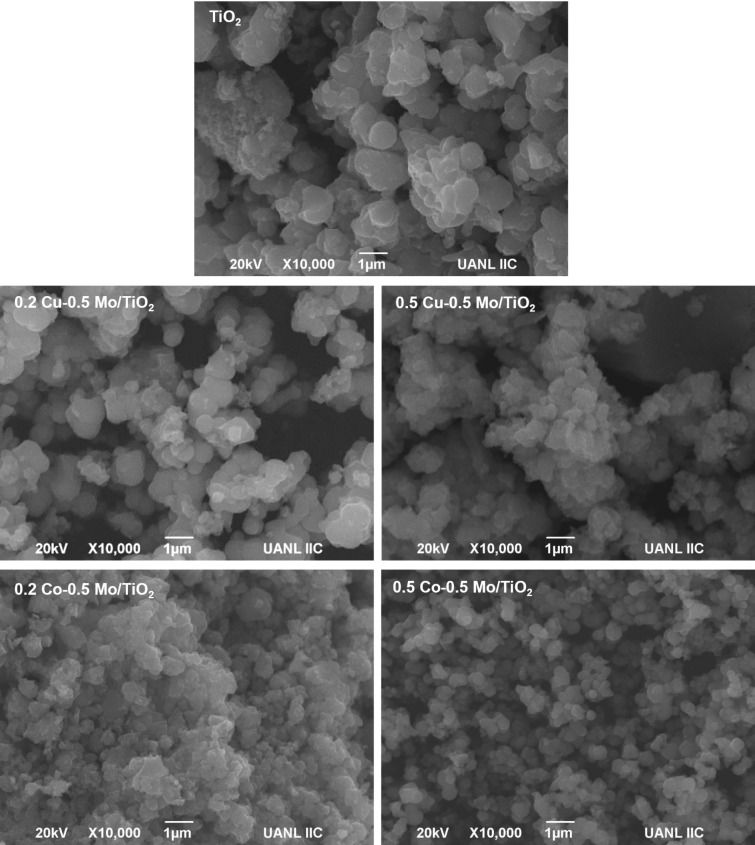
SEM images of TiO_2_ and co-doped TiO_2_ materials.

The morphological analysis results of co-doped TiO_2_ materials are shown in Figure 2. The titanium oxide prepared by the sol–gel method exhibits a poorly defined spherical morphology. With the incorporation of 0.2 wt % of copper and 0.5 wt % of molybdenum, a morphology quite similar to that of the pure oxide is observed.

The increase in copper concentration (0.5 wt %) resulted in a decrease in particle size and their agglomeration, leading to particles of undefined shape. In contrast, the incorporation of 0.2 wt % of cobalt resulted in smaller particles forming agglomerations. When the cobalt content is increased, particles with a better-defined spherical morphology and apparently smaller size than that of the pure oxide particles are observed.

The photocatalysts TiO_2_, Cu–Mo/TiO_2_, and Co–Mo/TiO_2_ were characterized through nitrogen physisorption analysis using the BET technique. Pure titanium oxide has a specific surface area of 34.4 m^2^/g; with the incorporation of 0.5 wt % Mo and 0.2 wt % Cu, the surface area decreases substantially to 16.4 m^2^/g. This result can be related to changes in the morphology and crystalline structure of the TiO_2_ after doping. The decrease in surface area after Cu doping is a behavior previously reported [17,32]. In contrast, with the addition of 0.5 wt % Mo and 0.2 wt % Co into TiO_2_, the area increases to 80.9 m^2^/g. These results are consistent with the morphological characterization, which showed a decrease in particle size in the cobalt-containing materials, which may have influenced the increase in specific surface area in these materials.

### Optical characterization

**Figure 3:**
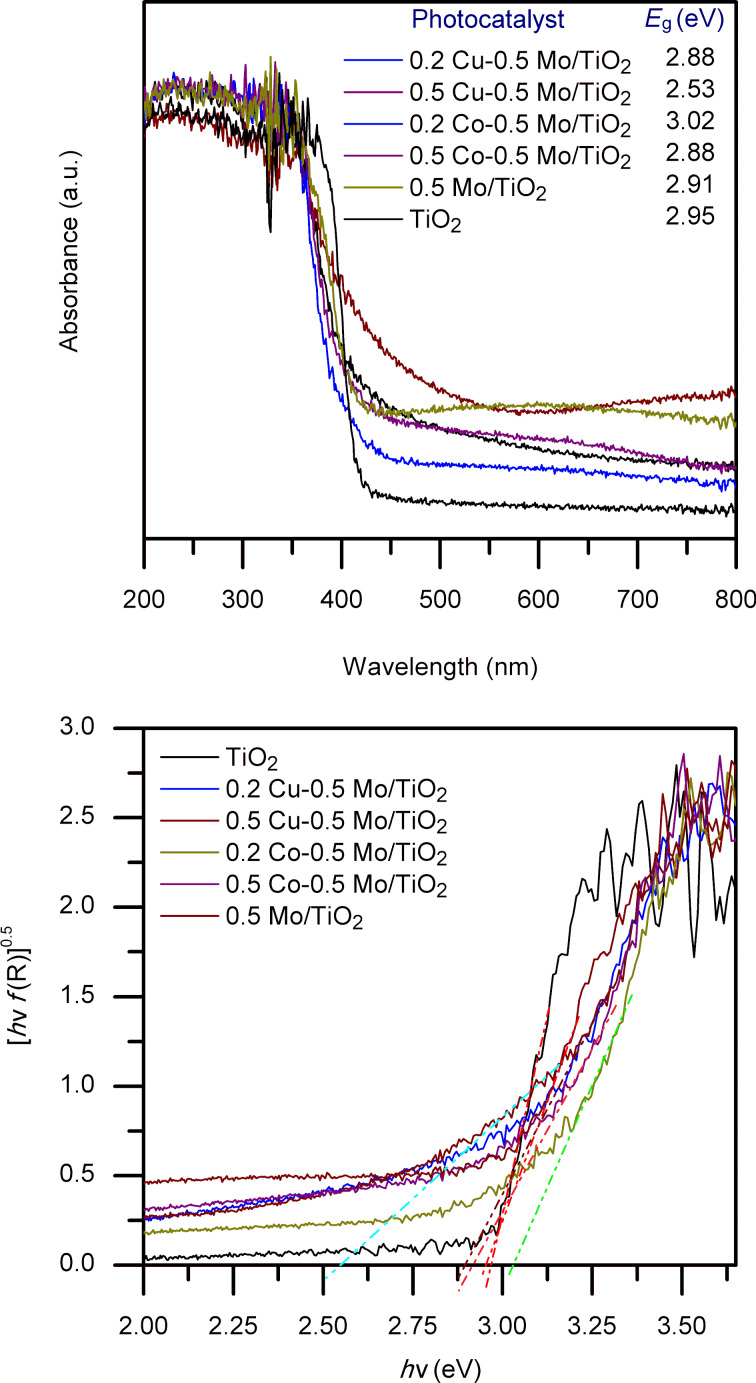
Diffuse reflectance UV–vis spectra of TiO_2_ and co-doped TiO_2_ materials.

The photocatalysts’ bandgap energy (*E*_g_) was measured by UV–vis diffuse reflectance spectroscopy. Figure 3 shows the absorption spectra of all the materials. Pure TiO_2_ absorbs light in the UV range. With the incorporation of 0.5 wt % of molybdenum, the photocatalyst’s absorption slightly increases toward the visible spectrum. Likewise, the incorporation of both copper and cobalt increases the absorption in the visible spectrum, which may be a consequence of the interaction of Mo–TiO_2_ with the copper and cobalt species. The bandgap energy values of the photocatalysts are reported in Figure 3. The improvement of light absorption by the photocatalysts can increase the production of photogenerated charges, which subsequently migrate to the catalyst’s surface, contributing to the efficient degradation of organic pollutants. The significant reduction in the bandgap energy as a result of copper incorporation is an effect previously reported in the literature as the introduction of Cu ions generates d orbitals below the conduction band, which reduces the bandgap energy [33–34].

**Figure 4:**
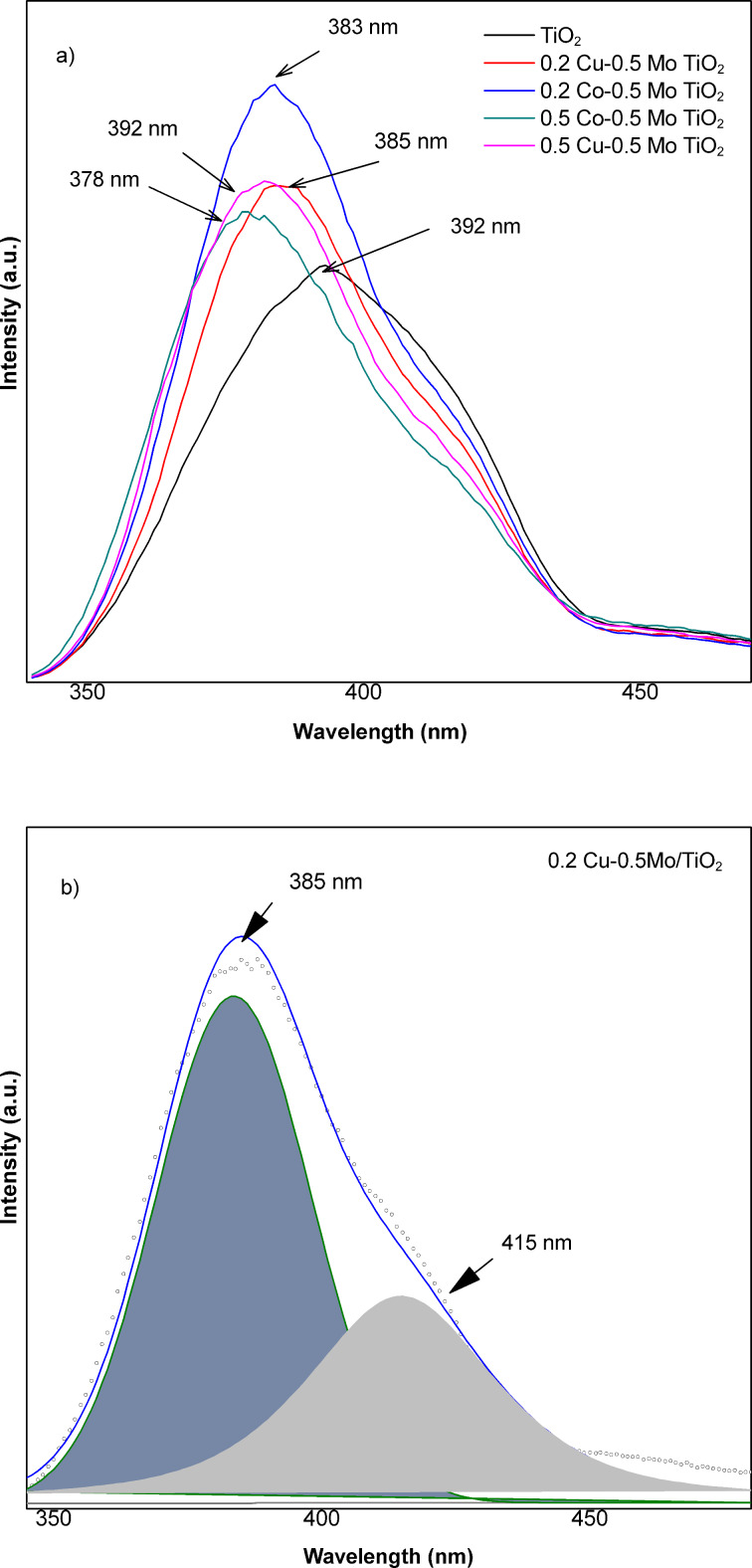
(a) Photoluminescence spectra of TiO_2_ and co-doped TiO_2_ materials and (b) deconvoluted photoluminescence spectrum of 0.2 Cu–0.5 Mo/TiO_2_ material.

To assess the efficiency of the separation of photogenerated charges in the photocatalysts, a photoluminescence analysis was performed. Figure 4 shows the photoluminescence spectra of pure titanium oxide and oxides containing Cu–Mo or Co–Mo obtained with an excitation wavelength of 310 nm. A broad signal centered at 392 nm is observed in the TiO_2_ spectrum, which can be assigned to band-to-band recombination [35], which may be caused by the interaction of electrons and holes present in the valence and conduction bands of TiO_2_ [36]. The photoluminescence spectra of co-doped materials show broad signals centered at 385 and 382 nm for materials with 0.2 and 0.5 wt % of copper, respectively, and at 383 and 378 nm, respectively, for materials with 0.2 and 0.5 wt % of cobalt. The signals generated by these photocatalysts were of higher intensity and showed a slight shift, which can be attributed to the formation of new energy levels within the bandgap of TiO_2_ [36]. The new energy levels can act as recombination centers for electrons and holes, affecting the emission and absorption of photons [37]. Doping metal ions such as Fe, Ni, Co, and Cu into TiO_2_ introduces mid-gap or surface states and defects, such as oxygen vacancies [38]. The deconvoluted spectrum of the 0.2 Cu–0.5 Mo/TiO_2_ material shows a new signal, which could indicate the generation of defects, which are generally related to the ability to capture electrons [36]. The PL intensity increase suggests a poor e^−^/h^+^ pair separation. So, it is possible that increased defect-related emission may coexist with trapping states. The relationships between PL intensity and photocatalytic activity are very complicated and depend on dopant species [39]. The inhibition of TiO_2_ phase transformation from anatase to rutile can increase surface oxygen vacancy and defect content [39]. Then, the inhibition of the rutile phase upon doping with Mo, as it was corroborated by XRD analysis, could affect the PL spectra. During the PL process, oxygen vacancies and defects can bind photoinduced electrons to form excitons so that PL can occur; the higher the content of surface oxygen vacancies and defects, the stronger the PL intensity [39]. Additionally, a signal was observed in the wavelength interval of 650–850 nm for all analyzed photocatalysts. The signals generated in the visible spectrum region (400–800 nm) are associated with oxygen vacancies, surface defects, and oxygen defects in TiO_2_ [36].

### Point of zero charge

**Figure 5:**
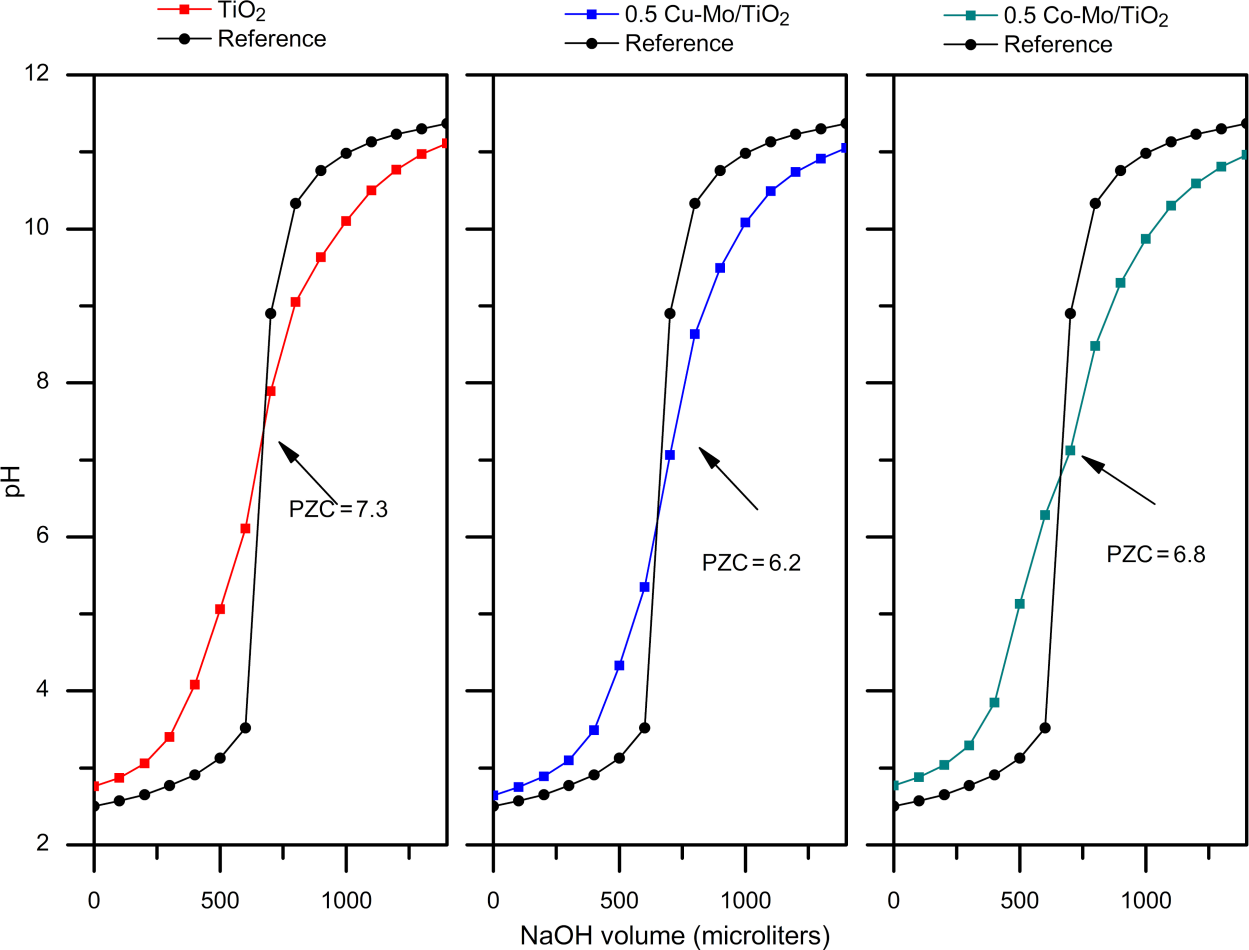
Point of zero charge of TiO_2_, Cu–Mo/TiO_2_, and Co–Mo/TiO_2_ catalysts determined by the acid–base titration method.

The point of zero charge (PZC) of the TiO_2_, 0.5 Cu–0.5 Mo/TiO_2_, and 0.5 Co–0.5 Mo/TiO_2_ photocatalysts was determined by the acid–base titration method [40]. Information on the surface properties of the catalysts is highly relevant as adsorption plays a vital role in photocatalytic activity. Figure 5 shows the titration curves for the analyzed samples. The PZC was determined at the intersection of the titration curve for the catalyst suspension and the reference curve. For pure titanium oxide, a PZC value of 7.3 was obtained. In contrast, for the materials containing copper and cobalt, the point of zero charge decreased, resulting in values of 6.2 and 6.8 for the catalysts 0.5 Cu–0.5 Mo/TiO_2_ and 0.5 Co–0.5 Mo/TiO_2_, respectively. The PZC value can be used to determine the optimal pH value of the ketoprofen aqueous solution for the photocatalytic reaction experiments. Since the photocatalysts are positively charged at pH < PZC, the electrostatic forces between the negatively charged organic molecules, such as the carboxylate groups of nonsteroidal anti-inflammatory drug molecules, and the positive charge of the catalyst surface, favor the interaction between both chemical species, which in turn may improve the subsequent photocatalytic reaction [41].

### Photocatalytic activity

**Figure 6:**
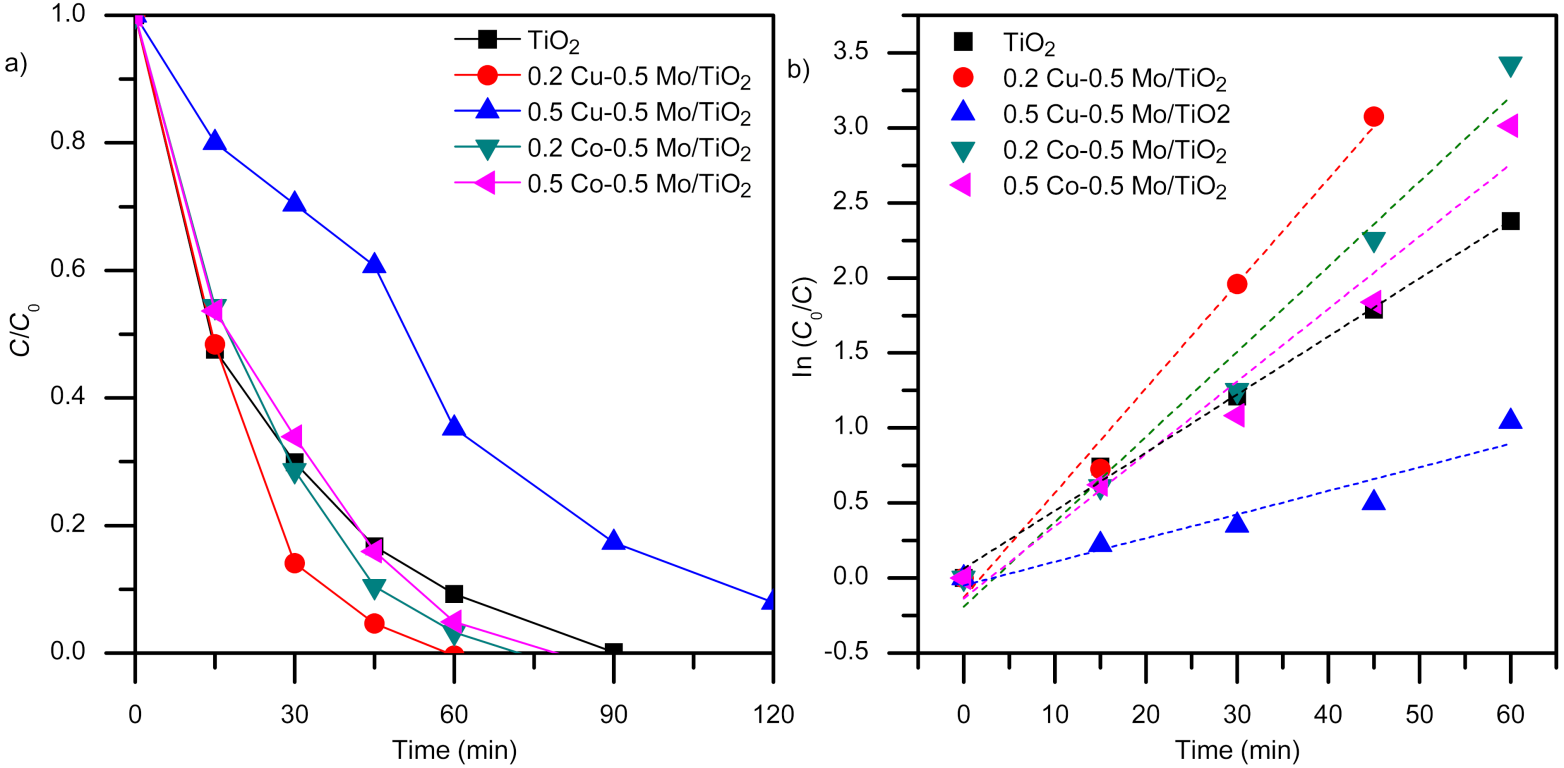
Photocatalytic degradation of ketoprofen using the co-doped TiO_2_. (a) Ketoprofen concentration profile, (b) pseudo-first-order plot (KTP initial concentration = 10 ppm, catalyst weight = 2 g·L^−1^, *V* = 200 mL, four UV lamps λ_max_ = 365 nm, O_2_ flow = 100 mL·min^−1^, analysis of reaction samples by HPLC).

Figure 6 shows the results of the ketoprofen degradation profile obtained by high-performance liquid chromatography (HPLC). The results indicate that ketoprofen is completely converted to its intermediate products within 90 min of reaction when pure TiO_2_ is used, whereas the conversion is achieved in 60 min with the most efficient photocatalyst, 0.2 Cu–0.5 Mo/TiO_2_. Numerous investigations have demonstrated that the photodegradation of organic pollutants follows a pseudo-first-order kinetic model [42–43]. The correlation between ln(*C*_0_/*C*) and irradiation time is shown in Figure 6, where the slope of the straight line for a pseudo-first-order process corresponds to *k*, the rate constant, which can be determined using the equation ln(*C*_0_/*C*) = *kt*. The apparent rate constants for each photocatalyst are reported in Table 2. These results indicate that the 0.2 Cu–0.5 Mo/TiO_2_ phototocatalyst with an *k*_app_ = 0.06974 min^−1^ is the most active, whereas 0.5 Cu–0.5 Mo/TiO_2_ with an *k*_app_ = 0.01574 min^−1^ is the least active. Figure 6 also shows that the Co–Mo/TiO_2_ materials degrade the drug more quickly than the pure oxide. However, although the 0.5 Cu–0.5 Mo/TiO_2_ photocatalyst promotes a significant reduction in ketoprofen concentration, complete degradation is not observed within 120 min of reaction. It has been reported that metal/metal co-doped photocatalysts can exhibit considerable selectivity in their catalytic efficiency towards different organic contaminant molecules [5]. The photochemical degradation of ketoprofen was previously reported [23] to result in a small decrease in the total organic carbon (TOC) content. TOC content remained almost constant for several hours, and only 12% of mineralization was achieved for an initial concentration of 100 ppm. During the photolysis experiments, ketoprofen is transformed into other aromatic compounds with similar structures. Table 2 shows the amount of ketoprofen adsorbed by the catalyst, *q*_s_, which is obtained by the mass balance: *q*_s_ = *V*/*W*(*C*_0_ − *C*_eq_), where *C*_0_ is the initial ketoprofen concentration, *C*_eq_ is the concentration at equilibrium, *V* is the volume of ketoprofen solution, and *W* is the weight of catalyst. The results indicate that the Cu–Mo/TiO_2_ photocatalyst adsorbs more ketoprofen, which could favor degradation. The nitrogen physisorption analysis previously described revealed a lower surface area for this material. However, the different surface charge properties obtained by the surface copper content, and confirmed by the evaluation of PZC could enhance the adsorption ability, improving the subsequent oxidation reaction.

**Table 2:** Photocatalytic oxidation of ketoprofen.^a^

Photocatalyst	*q*_s_ (mM_KTP_/g_cat_)	*k*_app_ (min^−1^)	*R* ^2^	ketoprofen mineralization (%)

TiO_2_	0.00083	0.03868	0.994	49
0.2 Cu–0.5 Mo/TiO_2_	0.00141	0.06974	0.984	90
0.5 Cu–0.5 Mo/TiO_2_	0.00466	0.01574	0.878	18
0.2 Co–0.5 Mo/TiO_2_	0.00065	0.05668	0.970	79
0.5 Co–0.5 Mo/TiO_2_	0.00091	0.04828	0.956	70

^a^Analysis of reaction samples by HPLC and TOC.

Table 2 shows the percentage of ketoprofen mineralization obtained after 6 h of reaction. The results indicate that the 0.2 Cu–0.5 Mo/TiO_2_ photocatalyst was the most efficient, not only in degrading but also in mineralizing the contaminant, achieving 90% conversion of ketoprofen to CO_2_ and water. The efficiency of the 0.5 Cu–0.5 Mo/TiO_2_ catalyst was considerably lower. The cobalt–molybdenum co-doped materials showed similar results; the material containing less cobalt was more efficient in mineralizing ketoprofen.

**Figure 7:**
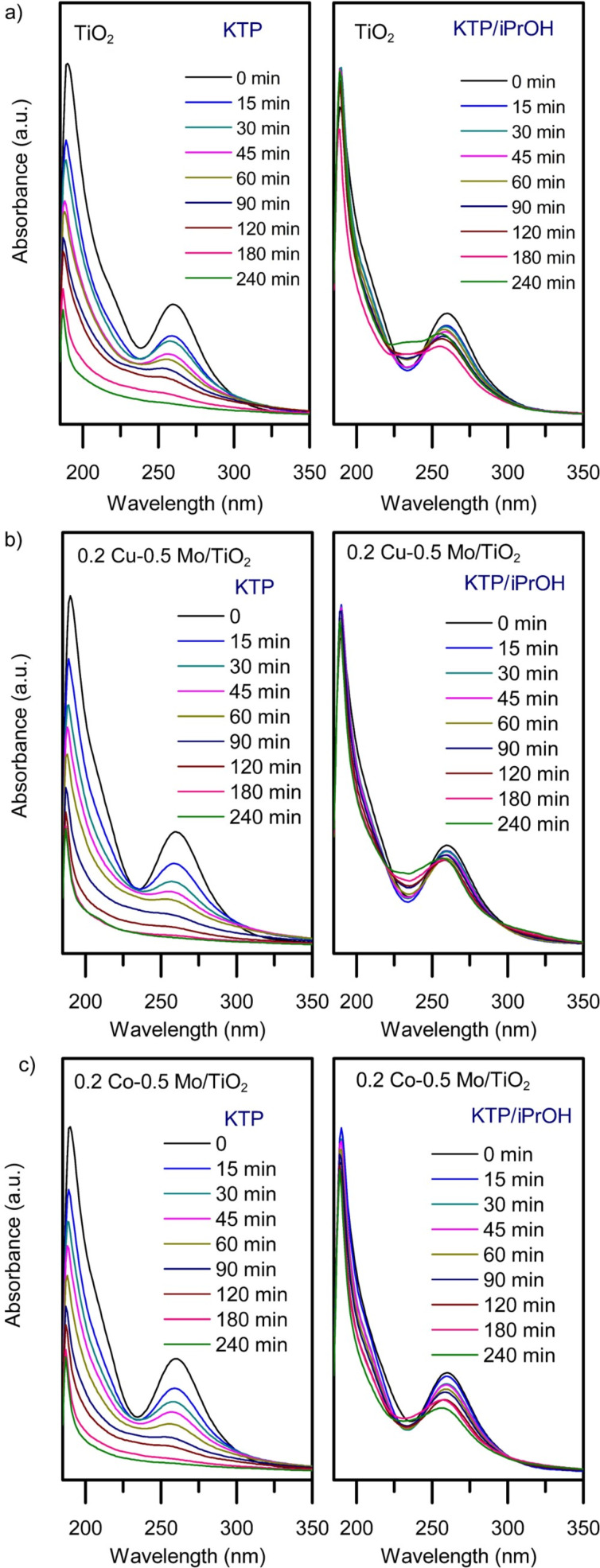
Ketoprofen degradation using (a) TiO_2_, (b) 0.2 Cu–0.5 Mo/TiO_2_, and (c) 0.2 Co–0.5 Mo/TiO_2_ as photocatalysts. KTP = without the addition of isopropanol and KTP/iPrOH = with added isopropanol. (KTP initial concentration = 10 ppm, isopropanol = 0.1 M, catalyst weight = 2 g·L^−1^, *V* = 200 mL, four UV lamps λ_max_ = 365 nm, O_2_ flow = 100 mL·min^−1^, analysis of reaction samples by UV–vis spectroscopy).

In a previous work, the ketoprofen degradation pathway using a TiO_2_ catalyst was reported [24]. According to intermediate organic products detected and the reaction route, it was determined that hydroxyl radicals, HO^·^, play the major role in the oxidation mechanism during the photodegradation reaction. To obtain more information about the photocatalytic oxidation mechanism of ketoprofen in this work, the importance of the presence of hydroxyl radicals generated by TiO_2_, Cu–Mo/TiO_2_, and Co–Mo/TiO_2_ catalysts was evaluated. Aliphatic alcohols (ROH) can be used as HO^·^ radical scavengers, to determine their effect on a photocatalytic process. The photocatalytic degradation reactions of ketoprofen solutions with an initial concentration of 10 ppm were carried out under the conditions as described in the Experimental section, but adding 0.1 M isopropanol (iPrOH). Figure 7 show the UV–vis spectra of ketoprofen obtained during the molecule’s oxidation reaction with and without the addition of alcohol, using pure TiO_2_, 0.2 Cu–Mo/TiO_2_, and 0.2 Co–Mo/TiO_2_. The results indicate that the presence of alcohol inhibited the photocatalytic oxidation of ketoprofen. Unlike the photocatalytic reaction carried out in the absence of the inhibitor, where the absorption bands of ketoprofen disappear after 90–180 min, when isopropanol is added, the intensity of the characteristic signals of the organic molecule persists for 240 min. The results of the photocatalytic activity evaluation of materials in the presence of alcohol confirm that in this research work hydroxyl radicals also play an important role in the oxidation of ketoprofen by the photocatalytic system; this effect is more pronounced with the Cu–Mo/TiO_2_ system.

## Conclusion

The synthesis of co-doped TiO_2_ materials with the combination of the metal ions Cu–Mo and Co–Mo was successfully carried out through the sol–gel method. Mo-doping stabilizes the anatase crystalline phase and distorts the TiO_2_ lattice structure. The introduction of the metallic ions Cu and Co was confirmed. Cu–Mo/TiO_2_ materials exhibited higher crystallinity, larger particle size, and lower surface area. However, the material 0.2 Cu–0.5 Mo/TiO_2_ was the most efficient in the photocatalytic oxidation reaction of ketoprofen, indicating that the composition, the light absorption improvement, the generation of surface oxygen vacancy and defects by the dopants, and the surface charge properties in this material enhance the photocatalytic oxidation of ketoprofen. Degradation of ketoprofen was complete after 60 min of reaction, and 90% of the total carbon was mineralized. In addition, it was determined that hydroxyl radicals play an important role in the photocatalytic reaction mechanism.

## Experimental

### Material synthesis

The synthesis of co-doped TiO_2_ was carried out through the sol–gel method. The reagents used were titanium butoxide (CAS: 5593-70-4, reagent grade 97%), ethanol (CAS: 64-17-5, reagent grade), ammonium molybdate tetrahydrate (CAS: 13106-76-8), copper nitrate (CAS: 10031-43-3), cobalt nitrate (CAS: 10026-22-9), cetyltrimethylammonium bromide (CTAB, CAS: 57-09-0), and acetic acid (CAS: 64-19-7)

TiO_2_ synthesis was carried out using a stoichiometric amount of the reactive CTAB dissolved in ethanol, then titanium butoxide was added dropwise. The solution was homogenized for 1 h. After that, in the hydrolysis step, a mixture of acetic acid, water, and ethanol was added dropwise, using a 1:10 molar ratio of Ti alkoxide/H_2_O to obtain the sol. The sol was kept under agitation for 2 h at 65 °C. The resulting gel was aged at room temperature for 24 h and dried at 60 °C for 12 h. Finally, the pure material was thermally treated following a heating program to 600 °C for 6 h. The synthesis of co-doped materials was carried out in a similar way.

For the Mo/TiO_2_ synthesis, a stoichiometric amount of the reactive CTAB was dissolved in ethanol, and then titanium butoxide was added dropwise. The solution was homogenized for 1 h. After that, ammonium molybdate tetrahydrate (0.5 wt %) and 0.5 mL of acetic acid were added to a mixture of deionized water and ethanol dropwise, using a 1:10 molar ratio of Ti alkoxide/H_2_O to obtain the sol. After the hydrolysis step, the sol was kept under agitation for 2 h at 65 °C. The resulting gel was aged at room temperature for 24 h and dried at 60 °C for 12 h. The incorporation of Cu and Co was achieved by impregnating titanium hydroxide. For this purpose, the dried titanium hydroxide powder was suspended in deionized water, and stoichiometric amounts of copper nitrate or cobalt nitrate were added to the suspension to obtain 0.2 wt % and 0.5 wt % of each dopant. The materials were kept under vigorous agitation for 2 h and then were recovered by filtration and dried at 60 °C. Finally, the material was thermally treated following a heating program to 600 °C over 6 h, with a controlled temperature increment. The photocatalysts obtained were labeled as 0.2 Cu–Mo/TiO_2_, 0.5 Cu–Mo/TiO_2_, 0.2 Co–Mo/TiO_2_, and 0.5 Co–Mo/TiO_2_.

### Characterization

The structural characterization was carried out using a Panalytical Empyrean diffractometer with Cu Ka radiation (λ = 1.5406 Å), scanning from 10° to 70°. The morphology was observed with a scanning electron microscope JEOL 6490LV equipped with an energy dispersive X-ray (EDX) spectroscopy analyzer for chemical microanalysis using 20 kV of voltage. The samples were placed on a carbon slab and covered with gold to improve the conductivity. The surface area was measured by N_2_ physisorption through the BET method using a NOVA 2000e Quantachrome Instrument. The XPS analysis was performed on a SPECS instrument with an energy analyzer, PHOIBOS 150 WAL. A UV–vis diffuse reflectance spectrophotometer, Thermo Scientific Evolution 600, was used to measure the photocatalyst bandgap energy (*E*_g_). All samples were analyzed in the range of 200–800 nm. The photoluminescence analysis was performed at room temperature with a fluorescence spectrophotometer Agilent Cary Eclipse, using an excitation wavelength of 320 nm. Peak deconvolution in the PL spectrum was done using the software XPSPEAK41 for deconvolution and extraction of data, using Gaussian peak fitting, and the software Origin Pro 8 to obtain the figures. The point of zero charge (PZC) of TiO_2_, Cu–Mo/ TiO_2_, and Co–Mo/ TiO_2_ was determined by the acid–base titration method [40]. For this purpose, 25 mL of a 0.1 M NaCl solution adjusted to pH 2.5 with 0.1 M HCl solution was placed in a 100 mL Pyrex glass flask and mixed with 0.3 g of the catalyst. The slurry was left agitating for 18 h. Then, the mixture containing the catalyst was titrated by adding 100 μL aliquots of a 0.1 M NaOH solution. The pH as a function of the volume of the NaOH solution added to the slurry was recorded. Separately, the same procedure was carried out without adding any catalyst.

#### Photocatalytic activity

The photocatalytic activity of the Cu–Mo/TiO_2_ and Co–Mo/TiO_2_ materials was evaluated in a custom-made reactor, equipped with a 400 mL Pyrex glass tube reactor and four long-wave UV-A lamps (15 W nominal power, Vilbert-Lourmat). The emission spectra extend from 300 to 600 nm, with three UV maxima at 352, 365, and 405 nm, and three additional peaks in the visible region at 436, 546, and 579 nm. This reactor system has previously been used to study the photocatalytic degradation of several aromatic organic compounds, such as phenol, acetaminophen, metoprolol, and diclofenac, using TiO_2_ Evonik P25 illuminated with UV light under a continuous flow of oxygen [41,44–45]. For each experiment, 200 mL of the ketoprofen aqueous solution was placed inside the Pyrex glass reactor and mixed with 2 g·L^−1^ of catalyst under dark conditions for 30 min to reach the adsorption–desorption equilibrium. At this time, the amount of ketoprofen adsorbed by each catalyst was evaluated by a mass balance. Then, the UV light lamps were turned on to induce the simultaneous formation of electron holes (h_vb_^+^) and electrons (e_cb_^−^) on the photocatalyst surface. The photocatalytic activity of the prepared materials was determined by the degradation of the ketoprofen aqueous solution with an initial concentration of 10 ppm. Samples were taken at different reaction times and analyzed by UV–vis spectroscopy, HPLC, and TOC measurements. Before analysis, all reaction samples were filtered through a 0.22 μm GV cellulose acetate membrane (Millipore Corp. Bedford, MA, USA). Chemical analysis of the reaction samples was carried out by UV–vis spectroscopy in a Shimadzu UV-2600 spectrophotometer. HPLC analysis of the reaction samples was carried out with a Thermo Scientific Surveyor instrument equipped with a photodiode array UV–vis detector. An Agilent Eclipse XDB-C-18 column (4.6 mm × 150 mm, 3.5 μm) was used to separate unreacted ketoprofen and intermediate organic reaction products. The mobile phase consisted of a mixture of acetonitrile and water 60:40 (v/v) acidified with 1% of acetic acid. The TOC content in these samples was measured with a Shimadzu carbon analyzer model 5000A. Photolysis experiments and degradation pathways were studied in previous works and already reported [23–24].

## Supporting Information

File 1 Additional XPS spectra.

File 2 Statistical analysis of reaction experiments.

## Data Availability

Data generated and analyzed during this study is available from the corresponding author upon reasonable request.
