# Supplementary material for: Synthesis of Cu–Mo/TiO2 and Co–Mo/TiO2 photocatalysts for the efficient degradation of organic pollutants in water
Source: Beilstein J Nanotechnol. 2026 Apr 27;17:559–70. doi: 10.3762/bjnano.17.37 (PMC13159265; doi:10.3762/bjnano.17.37)
Supplement: File 1 — Additional XPS spectra. [file Beilstein_J_Nanotechnol-17-559-s001.pdf]

BEILSTEIN JOURNAL OF NANOTECHNOLOGY

Supporting Information

for

Synthesis of Cu–Mo/TiO_2 and Co–Mo/TiO_2 photocatalysts for the efficient degradation of organic pollutants in water

Ilse Acosta, Brenda Zermeño, Edgar Moctezuma, Luis F. Garay-Rodríguez and Isaías Juárez-Ramírez

Beilstein J. Nanotechnol. **2026,** *17,* 559–570. doi:10.3762/bjnano.17.37

Additional XPS spectra

License and Terms: This is a supporting information file under the terms of the Creative Commons Attribution License (https://creativecommons.org/licenses/by/4.0). Please note that the reuse, redistribution and reproduction in particular requires that the author(s) and source are credited and that individual graphics may be subject to special legal provisions.
The license is subject to the *Beilstein Journal of Nanotechnology* terms and conditions: (https://www.beilstein-journals.org/bjnano/terms)

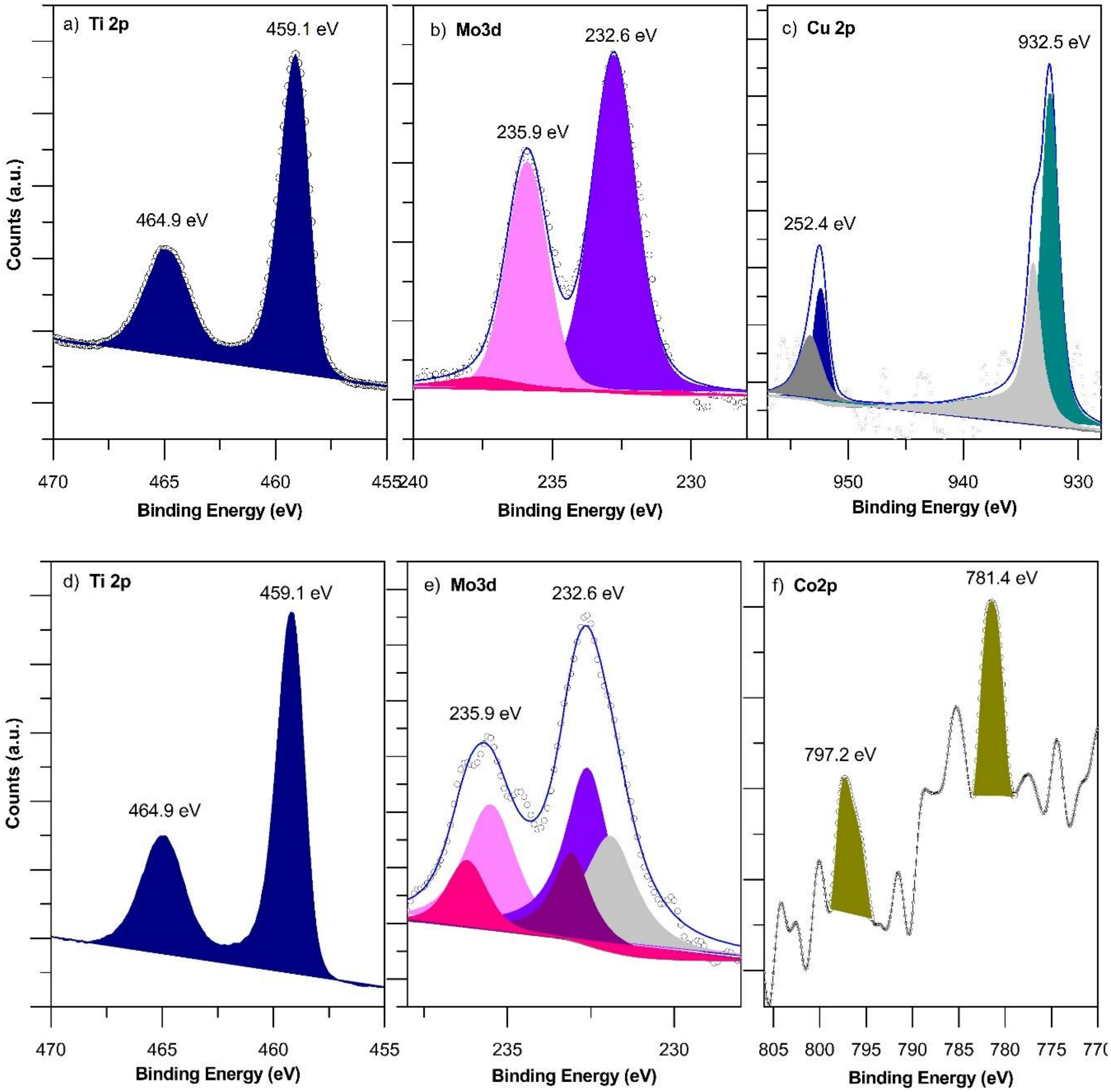

Figure S1: XPS spectra of a) Ti 2p spectrum of Cu–Mo/TiO_2, b) Mo 3d spectrum of Cu–Mo/TiO_2, c) Cu 2p spectrum of Cu–Mo/TiO_2, d) Ti 2p spectrum of Co–Mo/TiO_2, e) Mo 3d spectrum of Co–Mo/TiO_2, and f) Co 2p spectrum of Cu–Mo/TiO_2.
