# Supplementary material for: Synthesis of Cu–Mo/TiO2 and Co–Mo/TiO2 photocatalysts for the efficient degradation of organic pollutants in water
Source: Beilstein J Nanotechnol. 2026 Apr 27;17:559–70. doi: 10.3762/bjnano.17.37 (PMC13159265; doi:10.3762/bjnano.17.37)
Supplement: File 2 — Statistical analysis of reaction experiments. [file Beilstein_J_Nanotechnol-17-559-s002.pdf]

BEILSTEIN JOURNAL OF NANOTECHNOLOGY

Supporting Information

for

Synthesis of Cu–Mo/TiO_2 and Co–Mo/TiO_2 photocatalysts for the efficient degradation of organic pollutants in water

Ilse Acosta, Brenda Zermeño, Edgar Moctezuma, Luis F. Garay-Rodríguez and Isaías Juárez-Ramírez

Beilstein J. Nanotechnol. **2026,** *17,* 559–570. doi:10.3762/bjnano.17.37

Statistical analysis of reaction experiments

License and Terms: This is a supporting information file under the terms of the Creative Commons Attribution License (https://creativecommons.org/licenses/by/4.0). Please note that the reuse, redistribution and reproduction in particular requires that the author(s) and source are credited and that individual graphics may be subject to special legal provisions.
The license is subject to the *Beilstein Journal of Nanotechnology* terms and conditions: (https://www.beilstein-journals.org/bjnano/terms)

SD evaluated for the HPLC equipment and SD for photocatalytic experiments.

Table S1: Standard deviation evaluated for the HPLC equipment.

**Standard**	**Area**	**tr**
1	1133571	6.07
2	1137489	6.063
3	1117077	6.062
4	1130503	6.067
5	1135163	6.06
Average	1130760.6	6.0644
Deviation	8060.05749	0.00403733
SD	0.71279964	0.0665742

Table S2: Average SD obtained for the photocatalytic experiments evaluated by the TOC equipment.[a]

**Photocatalyst**	**Mineralization (%)**	**SD**
TiO_2	49	0.083
0.2 Cu–0.5 Mo/TiO_2	90	0.16
0.5 Cu–0.5 Mo/TiO_2	18	0.10
0.2 Co–0.5 Mo/TiO_2	79	0.113
0.5 Co–0.5 Mo/TiO_2	70	0.19

[a]The reaction experiments were carried out in duplicates and the analysis of reaction samples was carried out in triplicate.
